# Anti-Inflammatory Properties of Low and High Doxycycline Doses: An *In Vitro* Study

**DOI:** 10.1155/2015/329418

**Published:** 2015-04-22

**Authors:** Roberta Di Caprio, Serena Lembo, Luisa Di Costanzo, Anna Balato, Giuseppe Monfrecola

**Affiliations:** Department of Clinical Medicine and Surgery, Section of Dermatology, University of Naples Federico II, Via S. Pansini 5, 80131 Naples, Italy

## Abstract

Doxycycline is used to treat infective diseases because of its broadspectrum efficacy. High dose administration (100 or 200 mg/day) is often responsible for development of bacterial resistances and endogenous flora alterations, whereas low doses (20–40 mg/day) do not alter bacteria susceptibility to antibiotics and exert anti-inflammatory activities. In this study, we wanted to assess the efficacy of both low and high doxycycline doses in modulating IL-8, TNF-*α*, and IL-6 gene expression in HaCaT cells stimulated with LPS. Three experimental settings were used, differing in the timing of doxycycline treatment in respect to the insult induced by LPS: pretreatment, concomitant, and posttreatment. Low doses were more effective than high doses in modulating gene expression of LPS-induced proinflammatory cytokines (IL-8, TNF-*α*, and IL-6), when added before (pretreatment) or after (posttreatment) LPS stimulation. This effect was not appreciated when LPS and doxycycline were simultaneously added to cell cultures: in this case high doses were more effective. In conclusion, our* in vitro* study suggests that low doxycycline doses could be safely used in chronic or acute skin diseases in which the inflammatory process, either constantly in progress or periodically recurring, has to be prevented or controlled.

## 1. Introduction

Tetracyclines are a broad-spectrum antibiotics family active against a wide range of microorganisms including gram-positive and gram-negative bacteria, Chlamydiae, mycoplasmas, rickettsiae, and protozoan parasites [1, 2]. The parent compound chlortetracycline was first isolated from* Streptomyces aureofaciens* in 1947 [3]. Thereafter, other natural tetracyclines were isolated, including tetracycline, for which the family of molecules is named. Since then, the manipulation of natural antibiotics and the laboratory synthesis of novel compounds have enriched the tetracycline family. Compared with the original tetracycline, the synthetics, including minocycline and doxycycline, show a better pharmacokinetic profile than the first-generation tetracyclines when used orally, being rapidly and completely absorbed, even in elderly populations, with a longer half-life and excellent tissue penetration, with almost complete bioavailability [4, 5]. In addition to their well-characterized antibiotic effects, criticized in recent decades for the emergence of bacterial resistance [6], extensive research on tetracyclines has revealed a range of vastly important pharmacological properties [7] focused on the regulatory influence on the immune system and inflammatory pathway. The nonantibiotic anti-inflammatory effects of tetracyclines are under investigation for the treatment of multiple diseases. Their efficacy has already been reported in rheumatoid arthritis, corneal inflammation, gingivitis, osteoarthritic cartilage, allergen-induced inflammation, and a lot of other conditions like cardiovascular, neurological, and skin disorders [1, 7, 8]. The wide spectrum of anti-inflammatory effects ascribed to tetracyclines is probably due to their ability to interfere with the synthesis or activity of several mediators of inflammation. In addition, tetracyclines inhibit metalloproteinases (MMPs) and suppress hydrolases such as *α*-amylases and phospholipase A2 (key enzyme in the biosynthesis of inflammatory mediators such as the prostaglandins (PGE)) [7–9]. Furthermore, tetracyclines also scavenge reactive oxygen species and thereby prevent or reduce pathological tissue destruction [7]. Another important effect, significantly contributing to the overall immune-regulatory activity of tetracyclines, is the interference with cytokine production from neutrophils and macrophages during inflammatory conditions. Tetracyclines suppress cytokines, such as tumor necrosis factor-alpha (TNF-*α*), interleukin- (IL-) 1*β*, and IL-6, involved in inflammatory disorders as seborrheic dermatitis, psoriasis, acne, and rosacea [7–10]. For some of these diseases, principally, acne and rosacea, high doses of doxycycline (from 100 to 200 mg/die) are often administered, although they are frequently considered responsible for development of bacterial resistances and endogenous flora alterations. Concerning acne and rosacea treatment, quantitative bacteriologic studies demonstrate greater* in vivo* antimicrobial activity of minocycline compared with doxycycline and tetracycline against* Propionibacterium acnes* [11, 12]. Ishikawa et al. (2009) reported that minocycline reduces the protease-activated receptor 2-mediated production of IL-8 and thus attenuates the proinflammatory process in epidermal keratinocytes [13]. However, although minocycline is recommended for the aforementioned skin disorders, current therapy has moved towards the use of doxycycline, which has a similar efficacy for the treatment of these conditions with a reduced incidence of adverse effects, such as drug hypersensitivity syndrome, hyperpigmentation, and dizziness, frequently associated with minocycline therapy [8, 14, 15]. In recent clinical trials, lower doxycycline dose (40 mg/day) seems to be connected with the improvement of clinical manifestations in some dermatitis [10]. Low dose, administered once daily, appears to be effective and safe for the treatment of moderate to severe papulopustular rosacea, without bacterial flora alteration [10, 16]. The reason why this dose of doxycycline may produce this result is probably related to the specific involvement of lower doses of doxycycline in inflammatory mechanisms [17]. For this reason, aim of our study was to investigate,* in vitro*, if low doxycycline doses could be able to downregulate some of the cytokines involved in the pathogenesis of inflammatory skin diseases.

## 2. Materials and Methods

### 2.1. Cell Culture

Immortalized human keratinocytes (HaCaT) were grown in Dulbecco's modified Eagle's medium (DMEM, GIBCO, Grand Island, NY) containing 10% fetal bovine serum (FBS, GIBCO, Grand Island, NY), 2 mM L-glutamine (GIBCO, Grand Island, NY), and antibiotics (100 IU/mL penicillin G and 100 *μ*g/mL streptomycin, GIBCO, Grand Island, NY). Cells were cultured in a humidified incubator at 37°C with 5% CO_2_.

### 2.2. Treatment with Doxycycline

**Figure 1:**
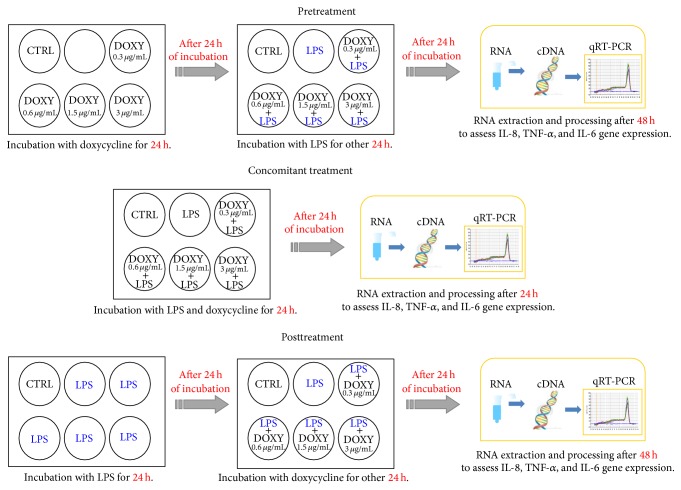
Study design. Schematic representation of the experimental settings with lipopolysaccharide (LPS) (25 *μ*g/mL) stimulation and doxycycline (DOXY) (0.3, 0.6, 1.5, and 3 mg/*μ*L) incubation. Times of stimulation with substances are described in detail in Section 2.3.

HaCaT cells were seeded onto 6-well culture plates in 2 mL of fresh culture medium until they reached a confluence of about 70%. After incubation for 1 day at 37°C in 5% CO_2_, cells were washed with phosphate-buffered saline (PBS, GIBCO, Grand Island, NY), covered with DMEM without serum, and stimulated with 25 *μ*g/mL of bacterial endotoxin-lipopolysaccharide (LPS) A (Sigma-Aldrich, Oakville, ON, Canada) in absence or presence of doxycycline (0.3 mg/*μ*L; 0.6 mg/*μ*L; 1.5 mg/*μ*L; 3 mg/*μ*L) (Sigma-Aldrich, Oakville, ON, Canada). These selected* in vitro* doses try to reproduce doxycycline plasma levels theoretically obtained after* in vivo* administration of 20, 40, 100, and 200 mg/day, respectively. Literature data indicate that the high doxycycline doses (100–200 mg/day) used to treat rosacea correspond to a plasma concentration of 1.5–3 mg/*μ*L, 2-3 hours after oral intake [6]. From that, we extrapolated the theoretical doxycycline blood concentrations of 0.3 and 0.6 mg/*μ*L obtained 3 hours after low doses (20–40 mg) oral assumption. To evaluate the protective or restoring doxycycline properties on LPS-stimulated HaCaT cells, three experimental settings were used, differing in the timing of doxycycline treatment in respect to the insult induced by LPS: pretreatment, concomitant, and posttreatment. For the pretreatment setting, cells were preincubated with doxycycline for 24 hours before receiving LPS; thereafter, 24 hours later, cells were harvested and mRNA extraction was performed: this was 48 hours after doxycycline incubation and 24 hours after LPS stimulation. In the concomitant setting, LPS and doxycycline were simultaneously added to the cells and mRNA was extracted 24 hours later. Lastly, for the posttreatment assessment, cells were stimulated with LPS for 24 hours and thereafter doxycycline was added; cells were incubated for further 24 hours and RNA extraction was performed at 48 hours (Figure 1). Cell viability was determined with the Trypan blue method after all treatments.

### 2.3. RNA Extraction and Real-Time

RNA was extracted (RNeasy Mini Protocol Qiagen, Valencia, CA) from HaCaT cells and cDNA was prepared according to the manufacturer's instructions. qRT-PCR (Transcriptor High Fidelity cDNA Synthesis, Roche, Indianapolis, IN) was used to analyze IL-8, TNF-*α*, and IL-6 gene expression. PCR primers were designed based on published sequences, and their specificity was verified with BLAST alignment search. The amount of mRNA for a given gene in each sample was normalized to the amount of mRNA of 18S reference gene in the same sample. Fold induction of gene expression was calculated using the ΔΔCT method as previously described [18].

### 2.4. Elisa

IL-8, TNF-*α*, and IL-6 protein levels were measured in supernatants from HaCaT cells stimulated or not with LPS (25 *μ*g/mL) at 24 and 48 h by single enzyme-linked immunosorbent assay (ELISA), using commercially available kits (R&D Systems, Minneapolis, MN, USA), according to the manufacturer's instructions.

### 2.5. Statistics

All statistical analyses were performed using GraphPad Prism 4.0 (GraphPad Software Inc., La Jolla, CA). Data that passed the normality test were analysed with two-tailed *t*-test. Values of *P* < 0.05 were considered significant. Data are expressed as means ± SD of three independent experiments, each performed in triplicate.

## 3. Results

**Figure 2:**
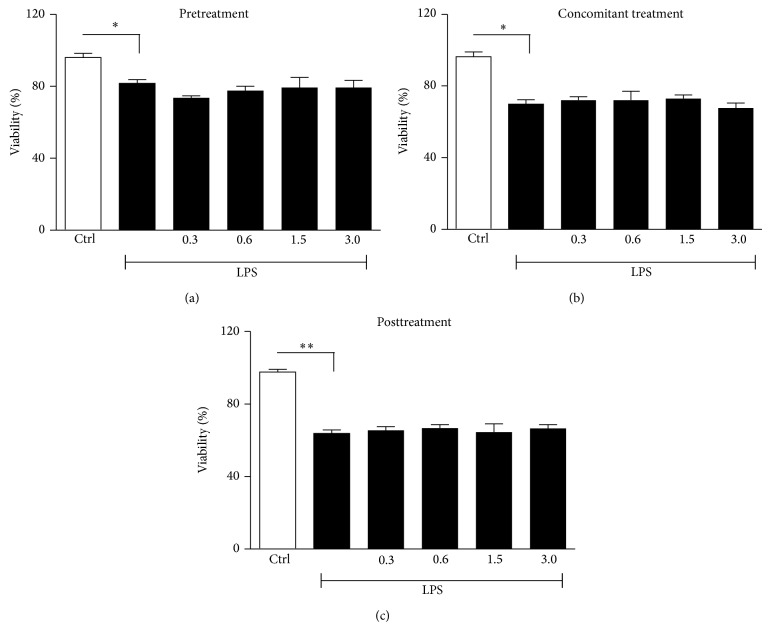
Evaluation of cell viability in HaCaT cells stimulated with LPS (25 *μ*g/mL) and incubated in pretreatment, concomitant, and posttreatment settings with low (0.3 mg/*μ*L; 0.6 mg/*μ*L) and high (1.5 mg/*μ*L; 3 mg/*μ*L) doxycycline doses. (a, c) Cell viability was measured at 48 h in pre- and posttreatment and (b) at 24 h in concomitant treatment. Statistical significance was determined with respect to the viability of nonstimulated cells (^∗^ *P* < 0.05, ^∗∗^ *P* < 0.01).

Doxycycline, at the four tested concentrations, did not exert any toxic effect toward HaCaT cells and after either 24 or 48 hours cellular viability results were similar to controls (data not shown). LPS, as expected, considerably reduced cell viability of 20, 28, and 36% in pretreatment, concomitant, and posttreatment settings, respectively. Doxycycline was unable to antagonize this toxic effect (Figure 2); nevertheless cell viability after LPS was considered sufficient to perform our experiments and to permit a correct analysis, as reported in previous investigations [19].

**Figure 3:**
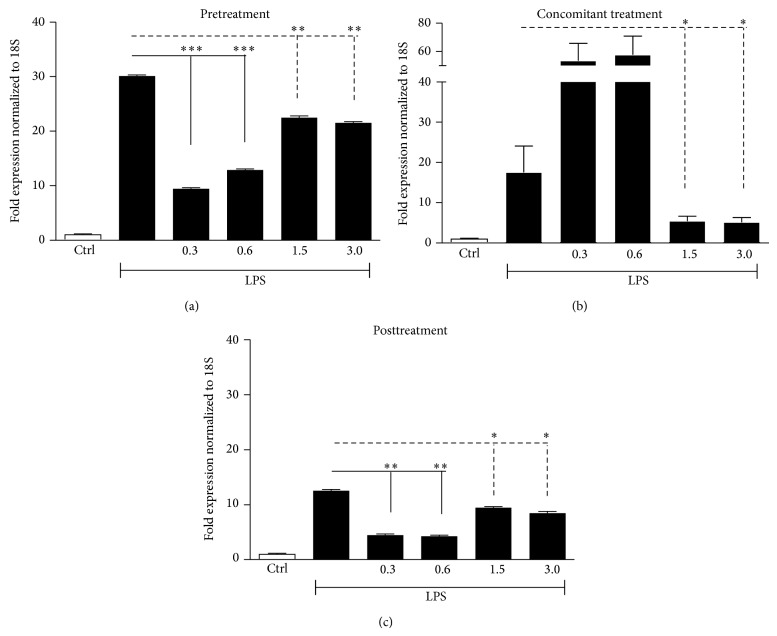
IL-8 gene expression, analyzed through qRT-PCR, in HaCaT cells stimulated with LPS (25 *μ*g/mL) and incubated in pretreatment, concomitant, and posttreatment settings with low (0.3 mg/*μ*L; 0.6 mg/*μ*L) and high (1.5 mg/*μ*L; 3 mg/*μ*L) doxycycline doses. IL-8 gene expression in (a) cells stimulated with LPS for 24 h and successively incubated with doxycycline for further 24 h, mRNA extraction performed at 48 h; (b) cells incubated with doxycycline immediately after LPS, mRNA extraction performed at 24 h; (c) cells pretreated with doxycycline for 24 h and successively stimulated with LPS; mRNA extraction at 48 h (^∗^ *P* < 0.05, ^∗∗^ *P* < 0.01, and ^∗∗∗^ *P* < 0.001; ns: not statistically significant).

**Figure 4:**
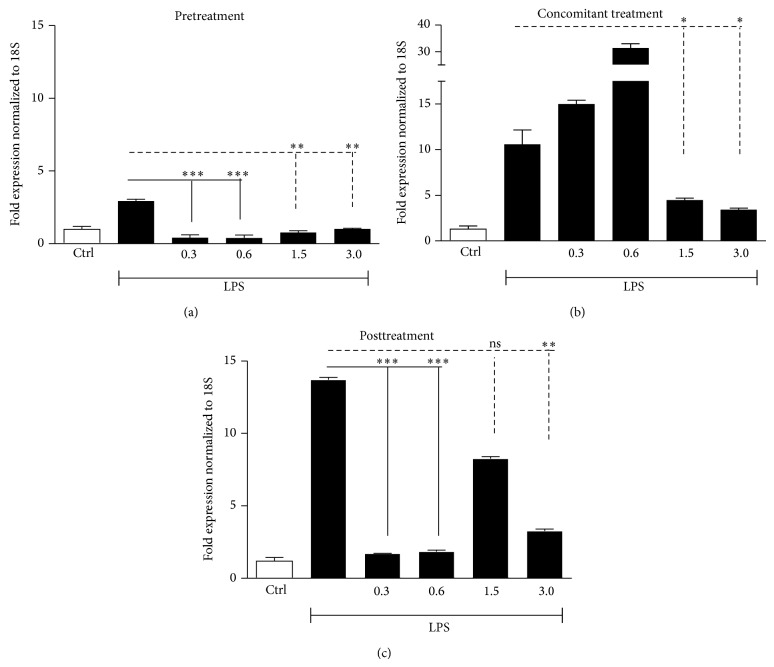
TNF-*α* gene expression, analyzed through qRT-PCR, in HaCaT cells stimulated with LPS (25 *μ*g/mL) and incubated in pretreatment, concomitant, and posttreatment settings with low (0.3 mg/*μ*L; 0.6 mg/*μ*L) and high (1.5 mg/*μ*L; 3 mg/*μ*L) doxycycline doses. TNF-*α* gene expression in (a) cells stimulated with LPS for 24 h and successively incubated with doxycycline for further 24 h, mRNA extraction performed at 48 h; (b) cells incubated with doxycycline immediately after LPS, mRNA extraction performed at 24 h; (c) cells pretreated with doxycycline for 24 h and successively stimulated with LPS, mRNA extraction at 48 h (^∗^ *P* < 0.05, ^∗∗^ *P* < 0.01, and ^∗∗∗^ *P* < 0.001; ns: not statistically significant).

**Figure 5:**
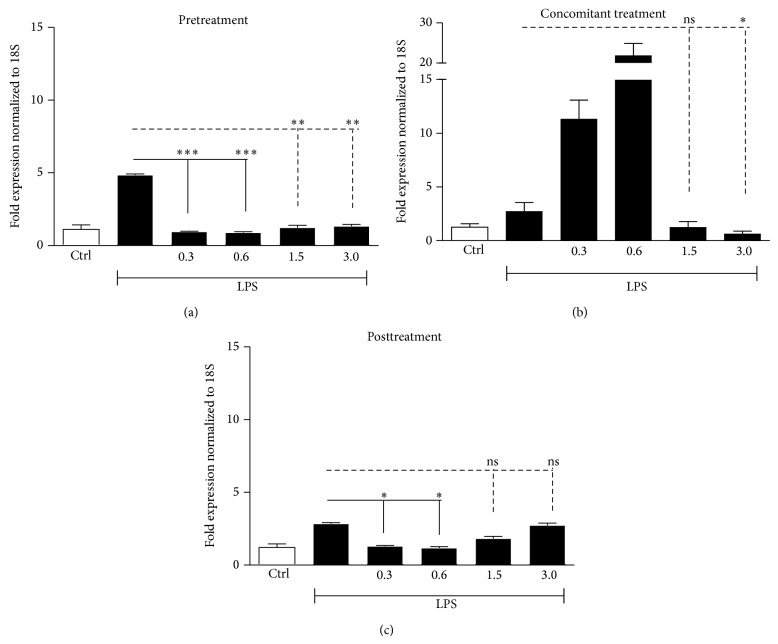
IL-6 gene expression, analyzed through qRT-PCR, in HaCaT cells stimulated with LPS (25 *μ*g/mL) and incubated in pretreatment, concomitant, and posttreatment settings with low (0.3 mg/*μ*L; 0.6 mg/*μ*L) and high (1.5 mg/*μ*L; 3 mg/*μ*L) doxycycline doses. IL-6 gene expression in (a) cells stimulated with LPS for 24 h and successively incubated with doxycycline for further 24 h, mRNA extraction performed at 48 h; (b) cells incubated with doxycycline immediately after LPS, mRNA extraction performed at 24 h; (c) cells pretreated with doxycycline for 24 h and successively stimulated with LPS, mRNA extraction at 48 h (^∗^ *P* < 0.05, ^∗∗^ *P* < 0.01, and ^∗∗∗^ *P* < 0.001; ns: not statistically significant).

**Figure 6:**
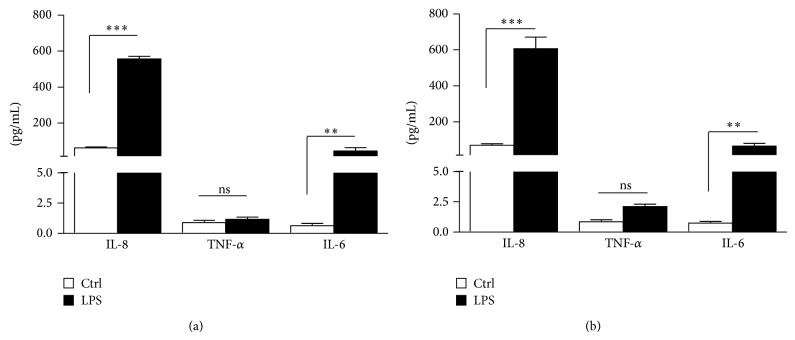
IL-8, TNF-*α*, and IL-6 protein levels in supernatants from HaCaT cells stimulated or not with LPS (25 *μ*g/mL) at (a) 24 and (b) 48 h (^∗^ *P* < 0.05, ^∗∗^ *P* < 0.01, and ^∗∗∗^ *P* < 0.001; ns: not statistically significant).

LPS enhanced mRNA levels of IL-8, TNF-*α*, and IL-6 in the three experimental settings (Figures 3, 4, and 5). These data were confirmed by the assessment of proteins in supernatants from HaCaT cells stimulated with LPS (25 *μ*g/mL) at 24 and 48 h (Figure 6).

Low doxycycline doses were more effective than high doses in modulating gene expression of LPS-induced proinflammatory cytokines (IL-8, TNF-*α*, and IL-6) when added before or after LPS stimulation (Figures 3–5, panels (a) and (c)). When LPS and doxycycline were simultaneously added to cell cultures high doses were more effective (Figures 3–5, panels (b)).

In particular, IL-8 increments, induced by LPS, showed a significant reduction (68.7 and 56.7%) in the pretreatment setting with low doxycycline doses (0.3 and 0.6 mg/*μ*L) (Figure 3(a)), as well as in posttreatment setting (64.3 and 66%) (Figure 3(c)). In a reduced manner, high doses were also able to decrease IL-8 levels in pretreatment setting (Figure 3(a)) and slightly significant differences were observed in posttreatment setting (Figure 3(c)). In regard to TNF-*α*, even if LPS induced a modest increase in the pretreatment setting, low doxycycline doses strongly downregulated its expression (86.4 and 86.7%), whereas high doses were able to induce a more moderate reduction (Figure 4(a)). Also in the posttreatment setting, TNF-*α* expression was better reduced by low doses (87.6 and 86.4%) whereas, of the two high doses, statistically significant effect was achieved only by doxycycline 3 mg/*μ*L (Figure 4(c)). IL-6 increments were reduced either by low or high doses in pretreatment setting (80.2 and 81.6%) (Figure 5(a)), whereas in the posttreatment setting significant differences were observed only for doxycycline 0.3 and 0.6 mg/*μ*L (Figure 5(c)). In the concomitant treatment, only high doxycycline concentrations were able to determine a statistically significant reduction in IL-8, TNF-*α*, and IL-6 expression (Figures 3(b), 4(b), and 5(b)), whereas low doses could not reduce inflammation but, apparently, enhanced the LPS-induced ILs increase.

## 4. Discussion

The rationale of the present study comes from the observation that the traditional antimicrobial doses of doxycycline can exert selection pressure, altering normal commensal microflora and increasing the risk of bacterial resistance [6–9, 20]. This “side effect” has not been detected, even during long-term treatment, using low doses of doxycycline (20–40 mg/day), able to exert their anti-inflammatory activities without altering bacteria susceptibility to antibiotics [16, 20, 21]. Herein, using immortalized keratinocytes stimulated with LPS, we provided evidence that both low and high doxycycline doses were able to modulate the expression of inflammatory mediators, in accordance with previous studies illustrating the capability of both anti-inflammatory and antimicrobial doses to be equally effective in rosacea treatment [16]. We have chosen HaCaT as cellular model because these cells are widely used in mechanistic and pharmacological studies and because they represent an easy way to handle substitutes for primary human keratinocytes. Recently, keratinocytes appear no longer as passive bystanders in the pathobiology of rosacea but actively contribute to inflammation and fibrosis specially by releasing proinflammatory cytokines, chemokines, growth factors, MMPs, and proteases [22–24]. Therefore keratinocytes are not only primary sensors of stressful conditions but also major players of the extremely complex immune response in the skin [25]. However, the exact role of keratinocytes in the context of rosacea still needs to be explored. In response to various external stimuli and/or to endogenous proinflammatory cytokines, epidermal keratinocytes release chemokines, such as IL-8, which recruit neutrophils and lymphocytes into the skin and exacerbate inflammation. Tetracyclines modulate this mechanism and attenuate the proinflammatory process in epidermal cells [26]. Several studies demonstrate that tetracyclines exert a range of nonantibiotic activities achieved at doses below the level needed for antibacterial ones, resulting in very interesting molecules for the treatment of a variety of noninfectious diseases, including rosacea. At present, doxycycline is the only tetracycline for which a dosage separation between antibiotic and anti-inflammatory effects has been established. Our findings regarding doxycycline capability to reduce cytokine production in the LPS-induced model inflammation agree with those of Yoshimura et al. [27] and Allon et al. [28], who demonstrated the beneficial effects of low doxycycline doses in several inflammatory diseases [29–31]. Interestingly, from our results, lower doxycycline doses were more effective than the higher ones, when added before or after LPS stimulation, but not in case of concomitant treatment when LPS and doxycycline were added simultaneously to cell cultures. Particularly, in our experiments, IL-8 increments showed a significant reduction in case of pretreatment with doxycycline 0.3 and 0.6 mg/*μ*L but also in case of posttreatment. Otherwise, high doxycycline doses (1.5 and 3 mg/*μ*L) were able to reduce IL-8 levels in the pretreatment setting, whereas no significant differences were observed in posttreatment setting. IL-8 is a potent neutrophil chemoattractant and activating factor produced by a variety of cells, including keratinocytes, and its production is also augmented by TNF-*α* [32]. Son et al. found that endogenous IL-8 mRNA expression was reduced by doxycycline treatment in a dose-dependent manner in pancreatic cancer cells (PANC-1) and that TNF-*α*-induced IL-8 mRNA expression was effectively blocked by 48 h of doxycycline pretreatment [33]. Further surveys have shown that low doxycycline doses downregulate proinflammatory cytokines, including TNF-*α* and IL-1*β* in multiple models [7–9]. According to these authors, it is unclear whether the mechanism for this effect is an indirect result of other anti-inflammatory actions or whether tetracyclines have a more direct effect on cytokines release. They suggest the existence of a posttranslational effect since levels of secreted cytokines were decreased, whereas there was no decrease in cytokine mRNA [34]. This is partially in contrast with our results: we proved that doxycycline significantly modulates TNF-*α* gene expression (Figure 4) but also IL-8 and IL-6 (Figures 3 and 5), and this would probably correlate with proteins modulation. In fact, the increased gene expression found in HaCaT cell after LPS is confirmed in our study by ELISA (Figure 6). With regard to IL-6 expression, we found that both low and high doses significantly affected the increase induced by LPS in doxycycline pretreatment, whereas significant differences were observed only for 0.3 and 0.6 mg/*μ*L concentrations in the posttreatment setting. Our data agree with Ikeda-Dantsuji et al. who reported that the production of IL-6 was suppressed by the minimum inhibitory concentrations of doxycycline in trachomatis-infected human fibroblast-like synovial cells (HFLS) or HeLa 229 cells immediately or early after infection [35]. IL-6 is a multifunctional cytokine, produced by monocytes and keratinocytes upon inflammatory stimulation, such as exposure to UV radiation and bacterial endotoxin [36], whose main functions include activation of lymphocytes and induction of an acute phase response [37].

Although low doses of doxycycline were more effective in the pretreatment and posttreatment settings, in the concomitant treatment analysis only the higher concentrations of doxycycline were able to produce a statistically significant reduction of IL-8, TNF-*α*, and IL-6 expression, whereas low doxycycline doses apparently enhanced the LPS-induced ILs increase. This effect could be explained by the cellular responses to the stress caused by the concomitant addition of two substances. Indeed, these cells have been weakened by culture with serum-free medium, so that it seemed that they were not able to distinguish between LPS and low dose doxycycline stimuli, sensing both of them as proinflammatory triggers. This hypothesis could explain the paradoxical results observed for the concomitant setting. Pastore et al., in a study on environmental and endogenous stresses to the skin as causative of cancer, demonstrated that plant polyphenols (Pps), known for their antioxidant activity, used in combination with UVA or LPS stimuli induced strong increase of stress responses in HaCaT [38]. Therefore, molecular responses of HaCaT to a combination of LPS and PPs showed synergism between the two factors in upregulation of proinflammatory cytokines gene and protein expression [38].

Probably, low doxycycline doses are more effective to prevent inflammation (pretreatment setting) or to reduce it if the process is already in progress (posttreatment), because they are rapidly absorbed and have a prolonged half-life, with potential preventive or antagonistic effect toward the damaging stimuli (LPS in our case). Inflammatory process usually involves three phases associated with different pattern: acute inflammation, immune response, and chronic inflammation. The acute inflammation characterizes the initial response to tissue injury and is related to the release of autacoids as histamine, serotonin, bradykinin, prostaglandins, and leukotrienes [39]. Acute inflammation presents a relatively short duration and may be followed by an immune response characterized by the activation of immune cells and by chronic inflammation. Thus, the mechanisms involved in the cellular and molecular pattern of acute and chronic inflammations are different. Although there are some reports on the efficacy of second generation tetracyclines on central inflammatory conditions [40–42], there are few reports regarding their anti-inflammatory effects in peripheral acute processes [43].

## 5. Conclusion

Our study provides* in vitro* evidence that both low and high doxycycline doses are able to directly modulate the expression of inflammatory mediators, in accordance with previous* in vivo* studies, highlighting the better efficacy of low doses in multiple settings. Thereafter, we suggest that low doxycycline doses could be safely used in case of chronic or acute inflammatory skin disease in which the inflammatory process, either constantly in progress or periodically recurring, has to be prevented or controlled.
